# Obg-like ATPase 1 regulates global protein serine/threonine phosphorylation in cancer cells by suppressing the GSK3β-inhibitor 2-PP1 positive feedback loop

**DOI:** 10.18632/oncotarget.6496

**Published:** 2015-12-07

**Authors:** Dong Xu, Renduo Song, Guohui Wang, Prince V.S. Jeyabal, Amanda M. Weiskoff, Kefeng Ding, Zheng-Zheng Shi

**Affiliations:** ^1^ Department of Translational Imaging, Houston Methodist Research Institute, Houston, TX 77030, USA; ^2^ Department of Surgical Oncology, The Second Affiliated Hospital, School of Medicine, Zhejiang University, Hangzhou, Zhejiang 310009, China

**Keywords:** cell signaling, GSK3beta, protein phosphatase 1, positive feedback loop, OLA1

## Abstract

OLA1 is an Obg family *P*-loop NTPase that possesses both GTP- and ATP-hydrolyzing activities. Here we report that OLA1 is a GSK3β interacting protein, and through its ATPase activity, inhibits the GSK3β-mediated activation of protein serine/threonine phosphatase 1 (PP1). It is hypothesized that GSK3β phosphorylates inhibitor 2 (I-2) of PP1 at Thr-72 and activates the PP1 · I-2 complex, which in turn dephosphorylates and stimulates GSK3β, thus forming a positive feedback loop. We revealed that the positive feedback loop is normally suppressed by OLA1, and becomes over-activated under OLA1 deficiency, resulting in increased cellular PP1 activity and dephosphorylation of multiple Ser/Thr phosphoproteins, and more strikingly, decreased global protein threonine phosphorylation. Furthermore, using xenograft models of colon cancer (H116) and ovarian cancer (SKOV3), we established a correlation among downregulation of OLA1, over-activation of the positive feedback loop as indicated by under-phosphorylation of I-2, and more aggressive tumor growth. This study provides the first evidence for the existence of a GSK3β-I-2-PP1 positive feedback loop in human cancer cells, and identifies OLA1 as an endogenous suppressor of this signaling motif.

## INTRODUCTION

Reversible protein phosphorylation, mediated by the opposing actions of protein kinases and phosphatases, is a fundamental mechanism for cell signaling involved in almost all cellular activities, and deregulation of this mechanism can cause pathological conditions, such as cancer [1–3]. Cell signaling systems consist of not only linear pathways but also complex networks within and between pathways in the forms of signaling cascades and feedback loops [4, 5]. Notable kinase cascades include the PI3K/Akt and MAP kinase pathways [6, 7], whereas feedback loops are best known for the phosphatase-mediated negative feedback regulation of kinase cascades, for example, the deactivation of MAP kinases by the dual-specificity phosphatase [8]. However, positive feedback loops between kinases and phosphatases are less well documented except for two proposed examples: the Cdc2/Cyclin B kinase complex-mediated positive feedback loop that activates the Cdc25 phosphatase and promotes mitotic entry [9], and the GSK3β-protein phosphatase 1 (PP1) positive feedback loop [10][11].

GSK3 is a multifunctional protein serine/threonine kinase with ∼100 protein substrates [12] that regulates diverse signaling pathways, ranging from glucose metabolism and protein synthesis to proliferation, differentiation, and survival [13][14]. For most of its substrates, phosphorylation by GSK3β leads to functional inhibition. On the other hand, PP1 is a major protein Ser/Thr phosphatase that mediates the dephosphorylation of ∼100 substrates, including those involved in regulating the cell cycle, metabolism, cell survival, and apoptosis [15, 16]. The catalytic subunit of PP1 (PP1c, or simply PP1) is regulated by two types of regulatory subunits: the inhibitory subunits that include inhibitor-1 (I-1) and inhibitor-2 (I-2), and the targeting subunits that bring PP1c to a specific substrate. In the cytosol I-2 forms a complex with PP1 [17]. This PP1·I-2 complex was initially named ATP-Mg-dependent phosphatase, because it can be reactivated by a kinase (F_A_, later renamed GSK3) in the presence of ATP and Mg [18]. GSK3 phosphorylates I-2 at Thr-72 and triggers conformational changes in the complex [19]. Interestingly, as demonstrated by Zhang et al., the GSK3β-activated PP1·I-2 complex could dephosphorylate GSK3β itself at Ser-9, resulting in a more active kinase [10]. This positive feedback loop between GSK3β and PP1 was further recognized by Szatmari et al. in rat forebrain neurons [11]. However, the detailed mechanisms that regulate this positive feedback loop, particularly a suppressive mechanism, are not fully understood. Without a suppressor, a positive feedback loop would eventually turn into an “on” switch [20]. To date, the GSK3β-PP1 positive feedback loop has not been elucidated in any cancer models, and it is currently unclear how it contributes to the cancer cell signaling network.

We report here the identification of a new endogenous regulator of the GSK3β-PP1 positive feedback loop: the Obg-like ATPase 1 [OLA1 (ENSG00000138430)]. OLA1 belongs to the Obg family and YchF subfamily of *P*-loop GTPases [21–23]. The OLA1/YchF proteins are highly conserved from bacteria to humans, and unlike other Obg family members, they bind and hydrolyze both ATP and GTP [24, 25][26]. We have previously demonstrated that OLA1 plays an important role in regulating multiple stress responses including oxidative stress [27] and heat shock [28]. More recently, we revealed that OLA1 suppresses protein synthesis and enhances the integrated stress response (ISR) by interacting with eIF2 and interfering with the formation of the eIF2-GTP-Met-tRNAi ternary complex [26]. In OLA1-knockdown (OLA1-KD) human breast cancer cells, the ISR signaling in response to cellular stresses is attenuated, as evidenced by reduced expression of ATF4, a surrogate marker of ISR [29], and hypophosphorylation of eIF2α. Interestingly, in the harvested OLA1-KD tumor tissues, we also observed over-activation of GSK3β, as reflected by decreased GSK3β phosphorylation at Ser-9 (GSK3β-p) and diminished expression of Snail, a downstream target of GSK3β. Hence we hypothesize that OLA1 may have a more profound involvement in protein Ser/Thr phosphorylation and/or dephosphorylation. Indeed, in the present study, OLA1 was found to bind GSK3β and block its phosphorylation of I-2, thus inhibiting the GSK3β-PP1 positive feedback loop. Therefore, downregulation of OLA1 leads to increased activity of PP1, decreased Ser/Thr phosphorylation in multiple phosphoproteins, and even more dramatically, increased dephosphorylation on a global scale. Moreover, our data suggest that the over-activation of the positive feedback loop is associated with I-2 dephosphorylation, and with a more aggressive tumor growth phenotype.

## RESULTS

### OLA1-knockdown cancer cells grow into larger tumors in two xenograft models

**Figure 1:**
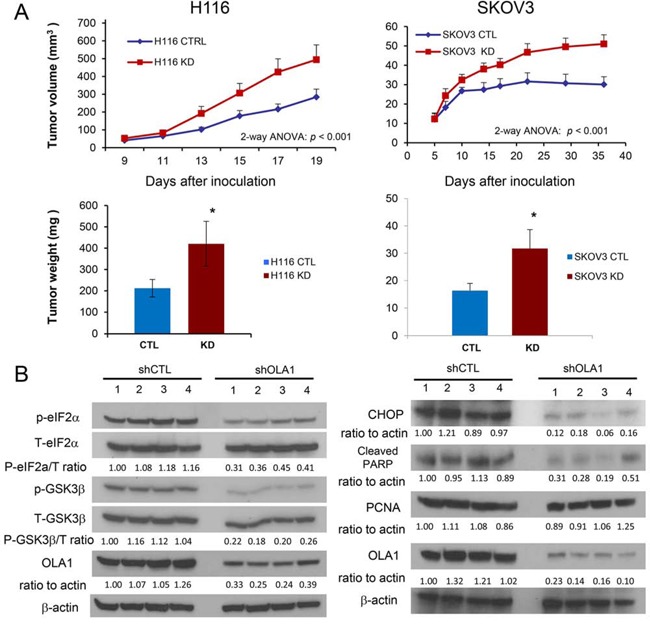
Knockdown of OLA1 promotes tumor growth *in vivo* Growth of xenograft tumors derived from H116 (left) and SKOV3 (right) cells stably transfected with control shRNA (CTRL) or OLA1 shRNA (KD). SCID mice were inoculated with the CTRL and KD cells on each side of their back, and the tumor growth was evaluated by measuring tumor sizes on the indicated days (top) and tumor weights (bottom) at the end of the experiment. Data are presented as mean ± SEM (*n* = 8/12 for H116/SKOV3 groups; **p < 0.001; two-way ANOVA). **B.** Immunoblot analysis of tumor tissues harvested from the xenograft models shown in A. Phosphorylation of eIF2α and GSK3β was assessed in the H116 tumors, and apoptotic and proliferative markers (CHOP, cleaved PARP, and PCNA) were evaluated in the SKOV3 model. For each blot, OLA1 was probed for verification of the knockdown, and β-actin for loading control; densitometric quantification was conducted using the NIH ImageJ software.

We recently reported that knockdown of OLA1 in breast cancer cells (MDA-MB231) reduces apoptosis without affecting proliferation, resulting in enhanced late-stage tumor growth [26]. In order to investigate the role of OLA1 in other cancer models, we established two new lines of OLA1-KD cells from the parental colorectal carcinoma (H116) and ovarian carcinoma (SKOV3) lines by stably expressing OLA1-specific shRNA (shOLA1), as well as the corresponding control shRNA transfected (shCTL) lines. When cultured *in vitro*, the OLA1-KD cells had a slightly lower rate of growth than the control cells (Figure S1). However, when inoculated into immunodeficient mice (SCID), OLA1-KD tumors of both H116 and SKOV3 origins showed significantly increased growth compared to the control tumors that were inoculated on the other side of the back of the same animal (Figure 1A). The average weight of the OLA1-KD H116 and SKOV3 tumors at harvest were 1.98 and 1.95 fold heavier than the control tumors, respectively. The harvested tumor tissues were analyzed by immunoblotting (IB) for possible molecular alterations. OLA1-KD tumors in the H116 colon cancer model showed markedly decreased eIF2α at Ser-51 (eIF2α-p) and reduced GSK3β-p, indicating hypoactive ISR and hyperactive GSK3 signaling (Figure 1B). Furthermore, in the OLA1-KD SKOV3 tumors, we found diminished expression of CHOP (a proapoptotic factor downstream of the ISR pathway) and reduced levels of cleaved PARP (an apoptotic marker), but no significant changes in PCNA (a proliferative marker). These data, in line with the previous report [26], confirmed that downregulation of OLA1 in human cancer cells enhances tumor growth *in vivo* and this effect is associated with decreased intratumoral apoptosis, attenuated ISR, and hyperactive GSK3β.

### OLA1 regulates serine/threonine phosphorylation in multiple phosphoproteins

**Figure 2:**
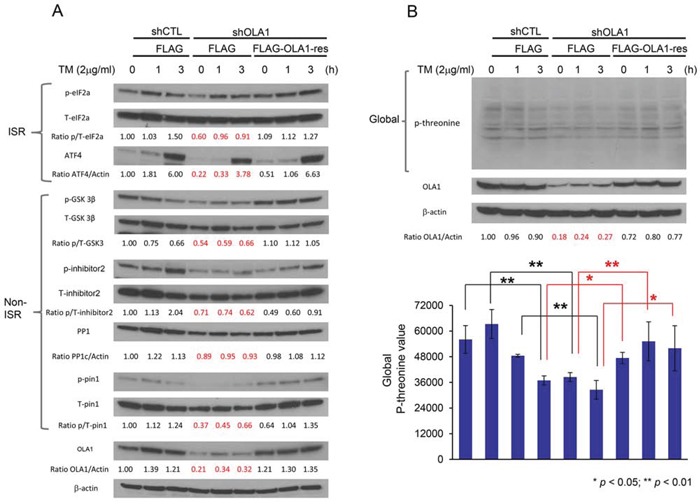
OLA1 negatively regulates protein Ser/Thr dephosphorylation **A.** Immunoblot analysis of key signal proteins in ISR and non-ISR pathways. A comparison among H116-derived cells: shCTL stably transfected and FLAG-only transiently transfected “control” cells, shOLA1 stably transfected and FLAG-only transiently transfected “OLA1-knockdown” cells, and shOLA1 stably transfected and FLAG-OLA1-res transiently transfected “OLA1-reconstituted” cells. Cultures were treated with 2 μg/ml tunicamycin (TM) for 0, 1, or 3 hours to induce ER stress. **B.** Immunoblot analysis of the total protein threonine phosphorylation. Experimental conditions are the same as “A” except that an anti-phospho-threonine antibody was used. Top: a representative immunoblot. Bottom: densitometric analysis of the immunoblots. Data are presented as means ± SD of p-threonine values (normalized by the loading control β-actin) from three independent experiments. *p < 0.05, **p < 0.01 (Student's t-test).

To explore whether OLA1 has a widespread role in protein phosphorylation through a mechanism independent of its interaction with eIF2 [26], we investigated protein phosphorylation in response to endoplasmic reticulum (ER) stress in two OLA1-KD cell lines (H116 and SKOV3). As shown in Figure 2A, tunicamycin (TM) induced eIF2α-p and expression of ATF4 in control H116 cells over time, however, the effect was weaker in OLA1-KD cells. At 3 h of treatment, the level of ATF4 in shOLA1 cells was ∼60% that of the control cells. Conversely, when the OLA1 levels were reconstituted in KD cells by transfection of an RNAi-resistant OLA1 cDNA construct, the “rescued” cells showed largely improved eIF2α-p and ATF4 induction. Next, GSK3β-p was evaluated. While there was no time-dependent phosphorylation of GSK3β because it is not a direct component of ISR, GSK3β-p was generally lower in OLA1-KD cells at all time-points than in CTL cells and recovered with OLA1 restoration (Figure 2A). Dephosphorylation of GSK3β-p indicates an active state of the kinase. When I-2, a substrate of GSK3β [19], was examined, OLA1-KD cells showed a substantially hypophosphorylated Thr-72 (Figure 2A). We also examined eIF2α, GSK3β, and I-2 in the second cancer line (SKOV3), and obtained the same results: they were all under-phosphorylated in the OLA1-KD cells as compared with the control cells (Figure S2). Moreover, we tested Pin1 (peptidyl-prolyl cis/trans isomerase) phosphorylation at residue Ser-16, which is not believed to be phosphorylated by GSK3β [30], [12]. Surprisingly, Pin1-p was also hypophosphorylated in the OLA1-KD H116 cells. We hence questioned if Ser/Thr residue hypophosphorylation was a global phenomenon in these cells. Using an antibody that recognizes universal threonine site phosphorylation, we confirmed that OLA1-KD cells globally exhibited lower Thr-p than both CTL cells (*p* < 0.01, for each time point) and OLA1-rescued cells (*p* < 0.01) (Figure 2B). Taken together, we believe that OLA1 regulates Ser/Thr phosphorylation of many proteins including and beyond eIF2α and GSK3β.

### OLA1-knockdown results in increased PP1 activity

**Figure 3:**
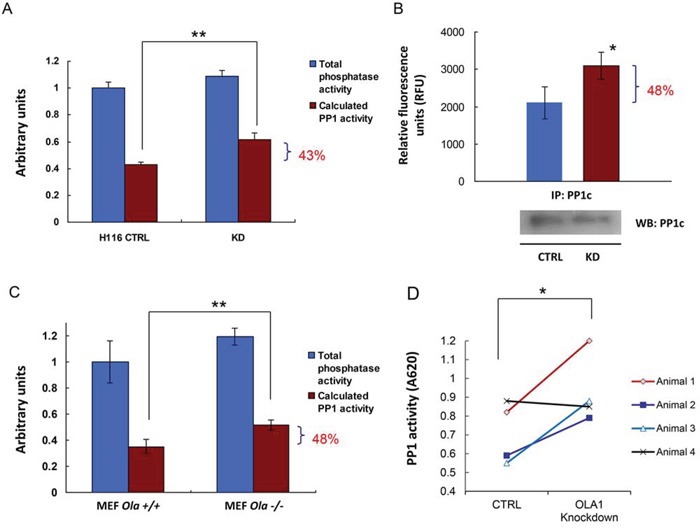
OLA1-knockdown cells have increased PP1 activity **A.** CTRL and OLA1-KD H116 cells were assayed for their PP1 activity using the malachite green assay. Data are presented as means ± SD (*n* = 3); **p < 0.01 (Student's t-test).**B.** PP1 was immunoprecipitated from CTRL and KD cells and measured for their protein phosphatase activities with the substrate of DiFMUP. Inset bottom: IB analysis of the IP products, which were used to normalize the PP1 activities. Data are presented as means ± SD (*n* = 3); *p < 0.05. **C.** PP1 assay using cell lysates prepared from the *Ola1*+/+ and −/− MEF lines. Data are presented as means ± SD (*n* = 3); **p < 0.01. **D.** PP1 assay using tissue extracts prepared from xenograft tumors derived from the SKOV3 CTRL or OLA1-knockdown cells. Paired data: the PP1 values obtained from the CTL and KD tumors on the same animal are connected by solid lines; *p < 0.05 (paired t-test).

The observed decrease in Ser/Thr phosphorylation of multiple proteins in OLA1-KD cells indicates that OLA1 may function to modulate the activities of kinases and/or phosphatases. OLA1 may interact with many kinases, phosphatases, or substrates individually. However, in the present study we focus on testing whether OLA1 acts through a unique molecular mechanism that triggers a shift of balance between Ser/Thr phosphorylation and dephosphorylation. Among the tested phosphoproteins (Figure 2A), eIF2α (Ser-51), GSK3β (Ser-9) [31], and I-2 (Thr-72) are confirmed substrates of protein phosphatase 1 (PP1) [32], and Pin1 (Ser-16) is a putative substrate for either PP1 or protein phosphatase 2A (PP2A) because Pin1-p is responsive to PP1/PP2A inhibitor Calyculin A (CA) [33]. Therefore, we questioned whether the OLA1-KD cells have increased PP1/PP2A activities. Because protein levels of PP1c were comparable between the control and OLA1-KD cells (Figure 2A), a malachite green-based PP1 assay was employed to measure the enzymatic activity [34]. As shown in Figure 3, The PP1 enzymatic activity from the total cell lysate of OLA1-KD H116 cells was 43% higher than that in CTL cell lysate, whereas the total Ser/Thr phosphatase activity showed no significant difference (Figure 3A). Further, PP1 protein was immunoprecipitated (IP) from the total cell lysates and subjected to the enzymatic assay. IP product from OLA1-KD cells was found to contain 48% more PP1 activity than that from the control cells (Figure 3B). Additionally, a pair of mouse embryonic fibroblast (MEF) lines carrying *Ola1*+/+ or −/− alleles was tested for their basal PP1 activities. Again, the knockout MEF showed a 48% increase in PP1 activity (Figure 3C). Finally, we analyzed SKOV3 xenograft tumor tissues and found that in 3 of 4 pairs PP1 was more active in the OLA1-KD tumor than the CTL tumor (Figure 3D).

### OLA1 binds with GSK3β and inhibits the GSK3-mediated reactivation of PP1

**Figure 4:**
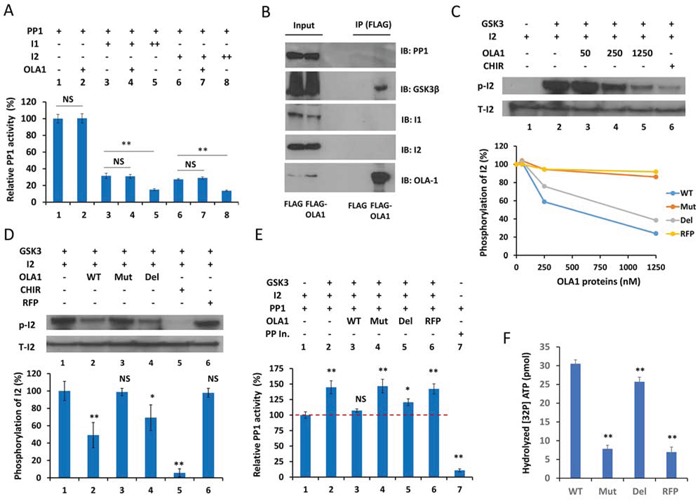
OLA1 inhibits PP1 reactivation by blocking GSK3β from phosphorylation of I-2 **A.** OLA1 does not directly inhibit PP1 activity or have an effect on the Inhibitor 1- or Inhibitor 2-mediated inhibition of PP1 (I-1 +: 10 ng/μl, ++: 20 ng/μl; I-2 +: 20 ng/μl, ++: 40 ng/μl; OLA1 +: 250 nM; incubation time: 30 min). PP1 activity was measured with the DiFMUP reagent. **B.** Co-IP of OLA1 with GSK3β but not with PP1, I-1, or I-2. **C.** OLA1 inhibits GSK3 activity in an *in vitro* I-2 phosphorylation assay. Top: immunoblot analysis shows that recombinant OLA1 WT protein decreases I-2 phosphorylation in a dose-response manner. The GSK3-specific inhibitor CHIR (2 μM) was used as the positive control. Bottom: dose-response curves of I-2 phosphorylation inhibition by OLA1 WT, N230A (Mut), and deltaTGS (Del) proteins, with RFP used as negative control. **D.** Quantitative analysis of I-2 phosphorylation inhibition by OLA1 WT and mutant proteins (250 nM, 1 hour). CHIR (20 μM) was used as a positive control and RFP was used as a negative control. **E.** OLA1 inhibits the GSK3β-mediated reactivation of PP1. GSK3β was added to the previously formed PP1 · I-2 complex, in which PP1 was inactivated to a basal level (designated here as 100%). Co-addition of OLA1 WT and Del proteins results in a total and partial blockage of the PP1 reactivation, respectively, whereas Mut and RFP proteins show no effect. All proteins were used at 250 nM for 1 hour. Protein phosphatase inhibitor (Roche) was used as a positive control. **F.** ATPase activity of OLA1 proteins. A ^32^P-γ-ATP-based assay was used to measure the ATP hydrolyzing activity of OLA1 WT and mutant proteins. RFP was used as a negative control. Data in all bar graphs are presented as means ± SD. *p < 0.05; **p < 0.01 (as compared with line 1 of each graph or between the bars as indicated; Student's t-test).

To interpret why OLA1-KD cells had increased cellular PP1 activity, we first tested whether OLA1 could directly inhibit PP1. In a simple *in vitro* protein phosphatase assay, human OLA1 protein failed to alter the dephosphorylation activity of the recombinant PP1c (Figure 4A, Lanes 1–2). OLA1 also did not enhance or suppress the inhibition of PP1 by I-1 or I-2 (Lanes 3–8). Because the purified inactive PP1 · I-2 complex can be reactivated by GSK3 through phosphorylation of I-2 [18][19], and a recent high-throughput affinity purification study indicated OLA1 as a GSK3β-associated protein [35], we investigated a possible role of OLA1 in GSK3-mediated PP1 reactivation. First, following IP of the ectopically expressed OLA1 in HEK293T cells, IB analysis showed that OLA1 indeed interacts with GSK3β, but not with I-1 or I-2 (Figure 4B). Second, recombinant OLA1 wild-type protein (WT) was found to inhibit the GSK3-induced phosphorylation of I-2 in a dose-response manner (Figure 4C). In the same assay, two mutant forms of the recombinant OLA1 protein were also tested: one containing a point mutation at the G4 motif of the G domain that abolishes nucleotide-binding (N230A) and the other a truncated protein with a deleted C-terminal TGS domain (ΔTGS). As compared with the WT, the ΔTGS showed slightly less inhibition of GSK3, whereas N230A nearly lost all inhibitory activity, similar to the control red fluorescent protein (RFP) (Figure 4C). A subsequent quantitative assay using equal concentrations (250 nM) of proteins confirmed that WT and ΔTGS inhibited GSK3 activity significantly, but N230A and RFP did not (Figure 4D). Third, OLA1 was able to block the GSK3-mediated reactivation of PP1 (Figure 4E). In the PP1 reactivation assay, recombinant GSK3 was added to the previously formed PP1-I-2 complex in the presence of Mg^2+^ and ATP, leading to a ∼40% increase in PP1 activity. Co-addition of OLA1 WT and ΔTGS proteins resulted in a total and partial blockage of the PP1 reactivation, respectively, whereas addition of N230A and RFP proteins showed no effect. These data strongly suggest that OLA1's inhibition of PP1 is a result of its inhibition of GSK3's phosphorylation of I-2 and reactivation of PP1, and this OLA1 activity requires a functional ATPase domain. Finally, using a ^32^P-γ-ATP-based ATPase assay, we confirmed that WT OLA1 protein possessed ATP hydrolyzing activity, whereas the ΔTGS and N230A proteins had a partial and total loss of the activity, respectively (Figure 4F).

### OLA1 serves as a suppressor of the GSK3β-I-2-PP1 positive feedback loop

**Figure 5:**
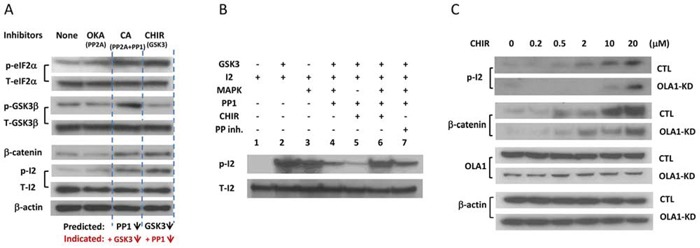
Validation of the GSK3-I-2-PP1 feedback loop and OLA1 as its suppressor **A.** Inhibitor studies in H116 cells that show linked changes of PP1 and GSK3β activities. Cells were treated with PP1 inhibitor Okadaic acid (OKA; 0.2 nM for 1 hour), PP1/PP2A inhibitor Calyculin A (CA; 2 μM for 0.5 hours) or GSK3-specific inhibitor CHIR-99021 (10 μM for 4 hours) and subjected to IB analysis with the indicated antibodies. Phosphorylation of eIF2α (Ser51) indicates the decreased activity of PP1, whereas the accumulation of β-catenin indicates the attenuation of GSK3β activity. β-actin was used as a loading control. **B.** *In vitro* I-2 phosphorylation assay explaining the hyper-phosphorylation of I-2 in response to both GSK3β and PP1 inhibitors. I-2 can be phosphorylated by both GSK3β (lane 2) and MAPK (lane 3) to form I-2-p, which can be dephosphorylated by PP1 (lane 4). This induction of I-2-p by CHIR (lane 6 vs. lane 4) is MAPK-dependent (lane 5 vs lane 6). A protein phosphatase inhibitor can restore the I-2-p (lane 7 vs. lane 4). **C.** Effect of OLA1 on the GSK3-I-2-PP1 positive feedback loop. H116 cells stably transfected with the control (CTL) and OLA1 shRNA (OLA1-KD) were treated with CHIR at the indicated concentrations for 6 hours and subsequently subjected to IB analysis with the indicated antibodies. Note that induction of I-2-p is less effective in the OLA1-KD cells than the CTL cells, and β-catenin accumulation is also attenuated in OLA1-KD cells.

**Figure 6:**
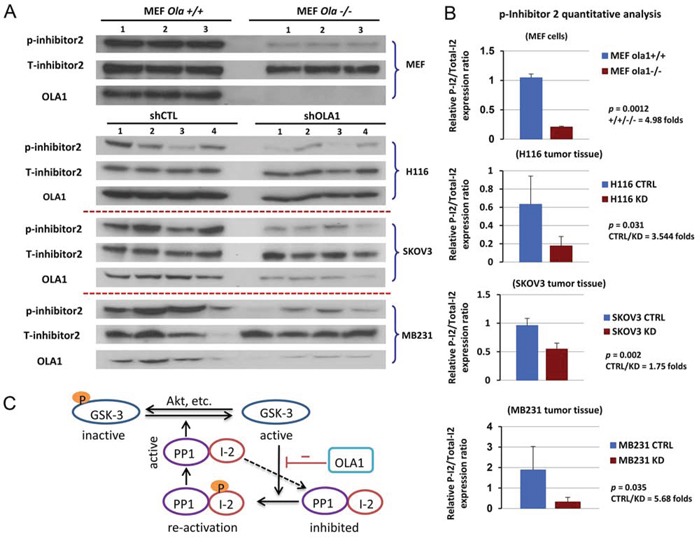
Hypophosphorylation of I-2 (Thr-72) is indicative of an activated GSK3-I-2-PP1 positive feedback loop *in vitro* and *in vivo* **A.** Immunoblot analysis of I-2 (Thr-72) phosphorylation level in *Ola1*+/+ and *Ola1*−/− MEF cells (top) and xenograft tumors derived from shCTL and shOLA1 cell lines of H116, SKOV3, and MB231 origins (bottom 3). **B.** Densitometric analysis of immunoblots shown in A. Data are presented as means of the ratios of I-2-p/total I-2 ± SD. Fold change in means between the control and knockdown groups, as well as *p* values (two-tail Student's t-test), are indicated for each bar graph. **C.** Model of the GSK3β-I-2-PP1 signaling circuit. PP1 (the catalytic subunit) is inhibited by its inhibitory subunit I-2; the complex can be reactivated through phosphorylation of I-2 by GSK3, and activated PP1 in turn dephosphorylates I-2 and GSK3, further promoting GSK3 activity. OLA1 functions as a suppressor of this loop.

A consequence of the reactivation of PP1 is the dephosphorylation of GSK3β, which further activates PP1. To demonstrate such a positive feedback loop, H116 cells were treated with protein phosphatase inhibitors [Okadaic acid (Oka) for PP2A, and CA for both PP2A and PP1], or a GSK3-specific inhibitor (CHIR-99021), and the resulting effects were evaluated with IB. As shown in Figure 5, both CHIR and CA inhibited GSK3β activity, as evidenced by the accumulation of its substrate β-catenin, whereas both inhibitors promoted the phosphorylation of eIF2α and I-2, indicating a suppressed PP1 (Figure 5A). Inhibition of PP2A alone (by 2 nM Oka), however, did not inhibit or activate PP1 or GSK3β. These inhibitor studies revealed the intrinsic linkage between PP1 and GSK3β activities, supporting the presence of a GSK3β-I-2-PP1 positive feedback loop. Moreover, we attempted to interpret a seemingly paradoxical effect of the feedback loop on I-2-p (Figure 5A) that was encountered in previous reports [10][11]: inhibition of either GSK3β or PP1 results in similarly increased Thr-72-p. In an *in vitro* system merely comprised of PP1, I-2, and GSK3, addition of CHIR could largely prevent the phosphorylation of Thr-72 (Figure 5B, Lane 5). Considering Thr-72 can be phosphorylated *in vivo* by other kinases including ERK1/2, MAPK, and CDKs [36], we reconstituted another system that included MAPK. CHIR instead promoted the formation of Thr-72-p in the presence of MAPK (Figure 5B Lane 6). Putting together these *in vitro* analyses and cell-based observations (Figures 2–4), we believe that Thr-72-p is an unambiguous indicator of the overall activity of the feedback loop, i.e., an increased Thr-72-p indicates the suppression of both GSK3β and PP1, and *vice versa*. To confirm this, we measured Thr-72-p of I-2 in the *Ola1*−/− MEFs that had shown increased PP1 activity (Figure 3C), and found they were deficient compared to the Ola1+/+ cells (Figure 6A).

Next, we investigated the role of OLA1 in the regulation of the GSK3β-I-2-PP1 positive feedback loop in H116 cells. The CTL and OLA1-KD cells were treated with various concentrations of CHIR and subjected to IB analysis to observe possible alterations of the positive feedback loop. As expected, the treatment induced Thr-72-p in both CTL and OLA1-KD cells in a dose-dependent manner. However, the OLA1-KD cells showed a severely attenuated phosphorylation response, indicating a sustained positive feedback loop, while the CTL cells did not (Figure 5C). Similarly, compared with the CTL cells, the OLA1-KD cells exhibited less β-catenin accumulation, indicating an increased resistance to GSK3 inhibition. These results suggest that OLA1 may function as a suppressor of the GSK3β-I-2-PP1 loop, and knockdown of OLA1 simultaneously confers increased PP1 and GSK3β activity.

Finally, tissue samples collected from three xenograft experiments were examined for I-2 phosphorylation, including the colon cancer (H116) and ovarian cancer (SKOV3) raised in the present study (Figure 1) and the previously established breast cancer (MDA-MB231) [26]. In all there three models, the OLA1-KD tumors showed significantly less I-2-p than the control tumors (Figure 6A–6B). These data suggest that OLA1 downregulation causes a hyperactive GSK3β-I-2-PP1 positive feedback loop *in vivo*.

## DISCUSSION

This study shows the first direct evidence for the existence of a GSK3β-PP1 positive feedback loop in a cancer signaling network, and presents a novel endogenous suppressor of that loop, OLA1. Our initial goal was to define the mechanisms underlying OLA1 KD-promoted tumor growth, which was initially discovered in a breast cancer model [26]. With the addition of two new cancer models, H116 colon cancer and SKOV3 ovarian cancer (Figure 1), we conclude that OLA1 plays a general inhibitory role in tumor growth *in vivo* while it has no effect or a slightly positive effect on cell growth *in vitro* [26] (Figure S1). Our interpretation is that the advanced tumor growth under OLA1-KD is not due to a change in cell proliferation, but a result of decreased apoptosis in response to intratumoral stresses, associated with attenuated ISR and reduced expression of CHOP (Figure 1B). More importantly, these studies suggested that OLA1 has another function: regulation of protein Ser/Thr phosphorylation (Figures 2–3).

We show that multiple specific phosphoproteins from ISR- and non-ISR pathways were hypophosphorylated in OLA1-KD cells, including eIF2α, GSK3β, I-2, and Pin1 (Figure 2A), and protein threonine phosphorylation was also globally decreased (Figure 2B). Conversely, when OLA1 expression was restored in OLA1-KD cells, phosphorylation of these sites recovered. In the literature, global changes in protein phosphorylation or dephosphorylation activity have been documented, though rarely. Reactive oxygen species have been shown to mediate a suppression of both protein tyrosine phosphatases and protein Ser/Thr phosphatases (PSP), accompanied by increased global protein phosphorylation, in skeletal muscle [37] and melanoma cells [38]. In contrast, in fibroblast cells infection of human cytomegalovirus mediates a rapid increase of cellular PSP [34]. In the present study, we show for the first time that knockdown or knockout of a *P*-loop NTPase (OLA1) causes imbalance of global protein Ser/Thr phosphorylation, by promoting the activity of PP1 (Figure 3).

To our surprise, OLA1 regulates PP1 activity not by directly inhibiting the phosphatase but by binding to GSK3β and blocking its phosphorylation of I-2 (Figure 4). Using reconstituted PP1 · I-2 complex, we confirmed that OLA1 inhibits the GSK3-mediated reactivation of the complex (Figure 4E). Previous studies have shown that GSK3 activity can be inhibited by proteins, such as the Wnt canonical pathway co-receptor LRP6 [39] and the A-kinase anchoring protein family member GSK3β interaction protein (GSKIP) [40], which binds and blocks GSK3 from phosphorylating its substrates. OLA1 represents a new class of GSK3 inhibitor that requires ATPase activity (Figure 4C, 4F). A simple assumption is that OLA1 competes with GSK3 for ATP and thus interferes with the phosphorylation reaction. Since only one substrate (I-2) was tested in our *in vitro* assays, it is unknown whether OLA1 generally inhibits GSK3 activity.

Based on our cell-based inhibitor tests and *in vitro* reconstitution assays (Figure 5), we propose here a positive feedback loop that consists of PP1, its inhibitor I-2, GSK3β, the kinase that inhibits the inhibitor, and additionally, an intrinsic suppressor for the entire loop – OLA1 (Figure 6C). When OLA1 is downregulated, this GSK3β-I-2-PP1 loop would stimulate the activities of both PP1 and GSK3β, resulting in a net decrease in protein Ser/Thr phosphorylation (Figure 2). From the viewpoint of stress-response dynamics, these cells may exhibit a right shift of the stimulation-phosphorylation curve, indicating a general desensitization [41]. This desensitization state is consistent with the previously observed attenuation of ISR [26]. Together, they provide a more comprehensive explanation, at the cell-signaling level, for the phenotypes demonstrated in OLA1-KD cells, including increased resistance to multiple cellular stresses [26], accelerated adhesion [42], decreased cell migration [43], and decreased apoptosis during tumor growth *in vivo*.

In our studies, we compared OLA1-KD cancer cells to control cells without comparison to any noncancerous cells. Therefore, the role of OLA1 or the positive feedback loop in oncogenic transformation is unknown. When several human cancers were compared to their normal tissue counterparts, OLA1 mRNA was actually increased in ≥50% of samples [44]. Based on analyses of 160 cases of breast cancer, we found that cancers with lower OLA1 protein expression have a worse prognosis than cancers with higher OLA1 levels, indicating that OLA1 plays a negative role in cancer progression rather than tumorigenesis [26]. Among the components of the positive feedback loop, GSK3β has been intensively studied for its role as either a tumor suppressor or promoter depending on the type of tumor [45]. GSK3 inhibitors have shown promising results in several preclinical anti-cancer studies [13]. It will be interesting to examine whether the effect of GSK inhibitors is linked to their function in interrupting the GSK3β-I-2-PP1 loop.

Another paradox exists regarding I-2 phosphorylation: increased GSK3β activity should produce more I-2-p; however, simultaneously, the activated PP1 should deplete I-2-p. We explored this using different *in vitro* and *in vivo* systems, and determined that I-2-p inversely correlates with the overall activity of the feedback loop (Figures 5–6). Because I-2 is a subunit of the PP1 holoenzyme, reactivated PP1 could instantly auto-dephosphorylate its I-2 subunit [46]. However, I-2 phosphorylation by GSK3β is a relatively transient event [19]. More importantly, of the three xenograft models tested (breast, colon, and ovarian), I-2-p was consistently lower in the OLA1-KD tumors (Figure 6A–6B). Therefore, low I-2-p may serve as an indicator of an activated GSK3β-PP1 positive feedback loop associated with a more aggressive growth phenotype. In summary, this report describes a novel role of OLA1 (an NTPase) in regulating the interplay between GSK3β (a kinase) and PP1 (a phosphatase), and reveals that OLA1 regulates the balance of protein Ser/Thr phosphorylation and dephosphorylation by suppressing the GSK3β-I-2-PP1 positive feedback loop, a novel signaling motif in the cancer signaling network.

## MATERIALS AND METHODS

### Cell culture

Human colon cancer cell line H116 and human ovarian cancer cell line SKOV3 were obtained from the American Type Culture Collection (ATCC). H116 cells were cultured in Dulbecco's Modified Eagle's medium (DMEM, Invitrogen, Carlsbad, CA, USA) supplemented with 10% fetal bovine serum (FBS, Thermo Scientific, Waltham, MA), 10 units/ml penicillin, and 10 mg/ml streptomycin, and SKOV3 cells were cultured in McCoy's 5A medium (Lonza, Walkersville, MD) supplemented with the same. Immortalized MEF lines were generated as previously described [29]. Cells were cultured at 37°C in a humidified atmosphere of 5% CO_2_.

### Antibodies, proteins and reagents

Antibodies against Phospho-GSK3β (Ser-9), GSK-3β, PP1c, β-catenin, Phospho-eIF2α(Ser-51), eIF2α, ATF4, CHOP, PCNA, and Phospho-threonine (P-Thr) antibodies were purchased from Cell Signaling Technology (Danvers, MA). Phospho-I-2 (Thr-72-p) and Phospho-Pin1 (Ser-16) antibodies were obtained from Abcam (Cambridge, MA). I-2 and Pin1 (total protein) antibodies were from R&D systems (Minneapolis, MN) and Santa Cruz Biotechnology (Dallas, TX), respectively. Anti-OLA1 and anti-β-actin antibodies were from Sigma-Aldrich (Saint Louis, MO). Peroxidase-linked secondary antibodies, including anti-mouse IgG and anti-rabbit IgG were from GE Healthcare (Pittsburgh, PA). Recombinant protein used in this study including I-2, PP1, and GSK-3 were purchased from New England BioLabs (Ipswich, MA). Active GSK3β was obtained from SignalChem (Richmond, BC, Canada). Recombinant HIS-tagged OLA1 proteins including OLA1-WT (NM_013341.3, 396 aa), OLA1-N230A, and OLA1-ΔTGS were custom-made by Epoch Life Science (Missouri City, TX). Okadaic acid and Calyculin A were purchased from Sigma-Aldrich, GSK3 inhibitor CHIR99021 from Calbiochem (EMD Millipore, Billerica, MA), and I-1 from Upstate (EMD Millipore).

### Cell transfection

Stable OLA1-KD cell lines were established using SMARTvector lentiviral shRNA particles (Thermo Scientific) as described in the previous report [26]. To reconstitute OLA1 expression, the OLA1-knockdown cells were transiently transfected with an OLA1-rescue vector (FLAG-OLA1-res) [26], in which the nucleotide sequence corresponding to the OLA1-shRNA (5′-AAGTATCTGGAAGCGAACA-3′) was modified to 5′-AAATACCTCGAGGCAAATA-3′ without changing the encoded amino acid sequence.

### Immunoblot analysis and IP

Immunoblot analysis was performed according to our standard procedures as described in earlier studies [27, 28]. For IP of FLAG-tagged proteins, anti-FLAG M2 magnetic beads (Sigma-Aldrich) were used according to the manufacturer's instructions as described [28].

### *In vivo* tumor growth models

The animal experiments were approved by the Institutional Animal Care and Use Committee at the Houston Methodist Research Institute. Female FoxChase SCID mice (beige, (CB17.Cg-Prkdc(scid)Lyst(bg-J)/Crl) purchased from Harlan (Indianapolis, IN) at ages of 8-10 weeks were used, with one group for the SKOV3 ovarian cancer model (*n* = 12) and another for H116 colon cancer (*n* = 9). To inoculate xenograft tumors, 1×10^7^ shCTL and 1×10^7^ shOLA1 cells were injected subcutaneously into the left and right back of each animal, bilaterally. Tumor growth was monitored twice a week. The tumor size was calculated by (L×W^2^)/2 where L is the longer axis of the measurements and W is the shorter axis.

### Phosphatase activity assays

For determination of endogenous PP1 activity, cultured cells or tumor tissue homogenates were extracted with phosphatase lysis buffer [47] containing 20 mM HEPES (pH 7.4), 10% glycerol, 0.1% Nonidet P-40, 1 mM EGTA, 30 mM b-mercaptoethanol, 1 mM phenylmethylsulfonyl fluoride, 2 μg/ml leupeptin, and 2 μg/ml aprotinin for 10 minutes at 4°C, and then subjected to centrifugation at 100,000×g for 10 minutes at 4°C. The resulting supernatant was passed through Sephadex G-25 (GE Healthcare, Pittsburgh, PA) columns twice by low-speed centrifugation (600×g, 5 minutes) to eliminate ATP and intracellular free phosphate. Total protein concentration of the phosphate-free cell extracts was determined using the Pierce BCA protein assay reagent. Activity of serine/threonine phosphatases was measured using a Malachite Green Phosphate Detection Solution (EMD Millipore) to detect the release of phosphate from a phosphopeptide substrate (RRA(pT)VA) [34]. PP1-specific activity can be calculated by including Oka (20 or 0.2 nM) in the assay, which distinguishes between PP1 and PP2A activities.

For *in vitro* PP1 assay, 20 units/ml of PP1 was incubated with 200 mM substrate 6,8-difluoro-4-methylumbelliferyl phosphate (DiFMUP, Invitrogen) in reaction buffer (50 mM HEPES, 100 mM NaCl, 1 mM MnCl_2_, 2 mM DTT, 0.01% Brij-35) in the presence or absence of OLA1 or control proteins. The reaction product was measured with a fluorescence microplate reader using 360 nm excitation and 460 nm emission filters.

### *In vitro* PP1 inhibition and reactivation assay

The PP1•I-2 complexes were prepared according to a previously described procedure [46]. Briefly, 20 U/ml PP1 prepared in the presence of Mn^2+^ was incubated with 20 mg/ml recombinant rabbit I-2 for 30–40 min at room temperature. The PP1•I-2 complexes were reactivated by addition of recombinant GSK-3 or p42 MAP kinase, with or without co-addition of an OLA1 or control protein. The reactions typically contained 50 mM Tris-HCl, pH 7.5, 0.1 mM EDTA, 0.125 mM ATP, 1.25 mM magnesium acetate, and 2 mg/ml GSK-3β or 1 mg/ml MAPK, and were incubated at 30°C for 10 min. The reaction mixtures were subjected to either the DiFMUP-mediated PP1 assay described above, or immunoblot analysis for levels of I-2-p.

### *In vitro* I-2 phosphorylation assay

Recombinant GSK-3β (2 mg/ml) was used to phosphorylate I-2 (20 mg/ml) in the presence of ATP and magnesium acetate (1× NEBuffer for Protein Kinases, New England BioLabs) at 30°C for 10 min. To test the effect of OLA1 proteins on this reaction, an OLA1 or control protein was added with the GSK-3β at the indicated concentrations. The reaction mixtures were analyzed by immunoblotting for I-2 phosphorylation.

### ATPase assay

To determine the ATP hydrolyzing activities of OLA1, 1 μM protein was incubated with [g-^32^P]ATP (3.3 nM, 0.5 μCi) in the presence of 100 μM of the unlabeled nucleotide in a 50 μl reaction buffer (25 mM HEPES-KOH pH 7.5, 80 mM potassium acetate, 2.5 mM magnesium acetate, 1 mM DTT) at 35°C. After 30 min, the reaction was stopped by the addition of 200 μl activated charcoal suspension (100 mg/ml Norit A® charcoal (Sigma-Aldrich) in 1N HCL). After centrifugation twice at 100,000×g, release of [^32^P] phosphate in the supernatant was measured by scintillation counting.

### Statistics

For *in vitro* studies, a two-tailed Student's *t*-test was carried out. For *in vivo* animal model studies, a two-way ANOVA was performed. Values of *p* < 0.05 were considered to be significant.

## SUPPLEMENTARY FIGURES AND TABLES


