# Supplementary material for: Obg-like ATPase 1 regulates global protein serine/threonine phosphorylation in cancer cells by suppressing the GSK3β-inhibitor 2-PP1 positive feedback loop
Source: Oncotarget. 2015 Dec 7;7(3):3427–39. doi: 10.18632/oncotarget.6496 (PMC4823117; doi:10.18632/oncotarget.6496)
Supplement: Supplementary file 1 [file oncotarget-07-3427-s001.pdf]

SUPPLEMENTARY FIGURES

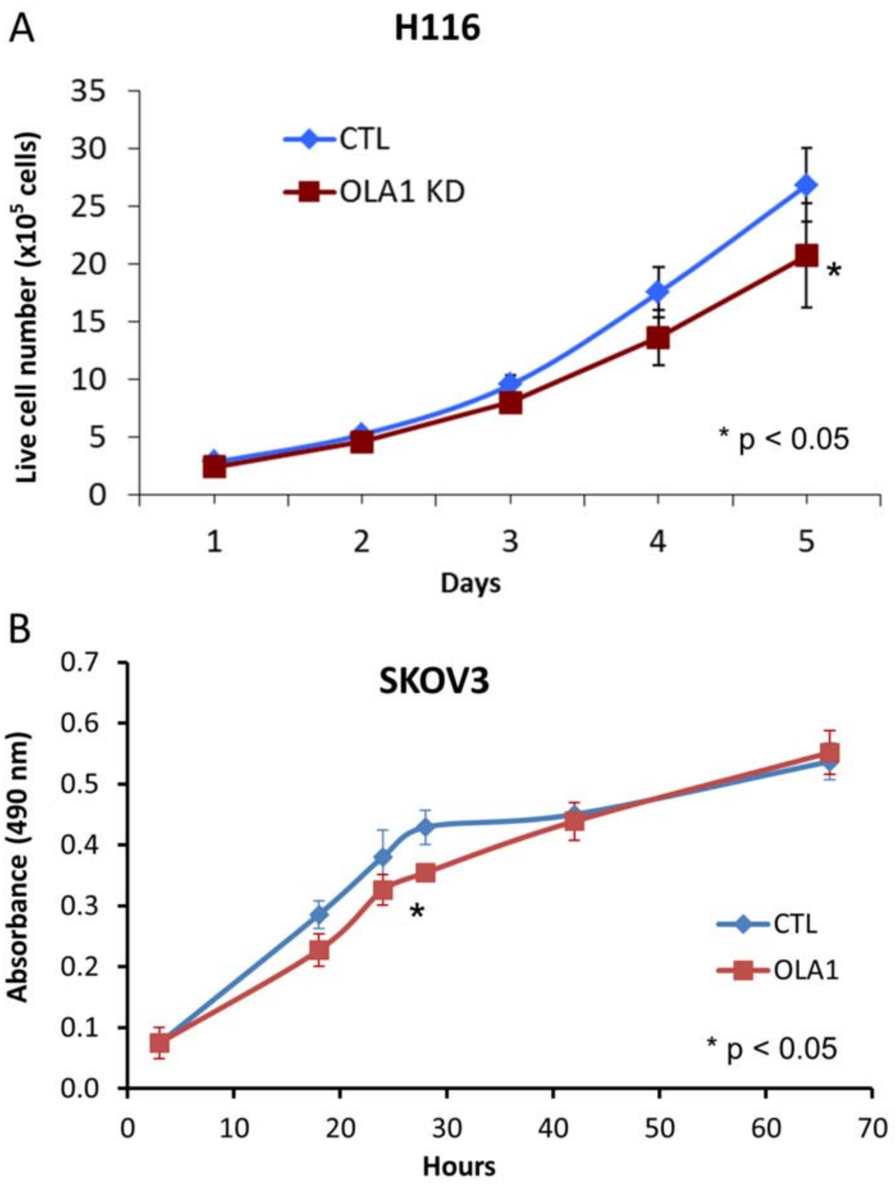

Supplementary Figure S1: Growth of OLA1-knockdown cancer cells *in vitro*. A. Human colon cancer cells (H116) were stably transfected with control shRNA and OLA1-specific shRNA to establish the CTL and OLA1-KD sublines, respectively. Cell growth was evaluated by Trypan blue assay on five consecutive days. **B.** Human ovarian cancer cells (SKOV3) were transiently transfected with control or OLA1-specific siRNA, and the cell growth was evaluated by MTS assay at the indicated time intervals. Data are presented as means ± SD (n = 3). *, $p < 0.05$ (Student's t-test).

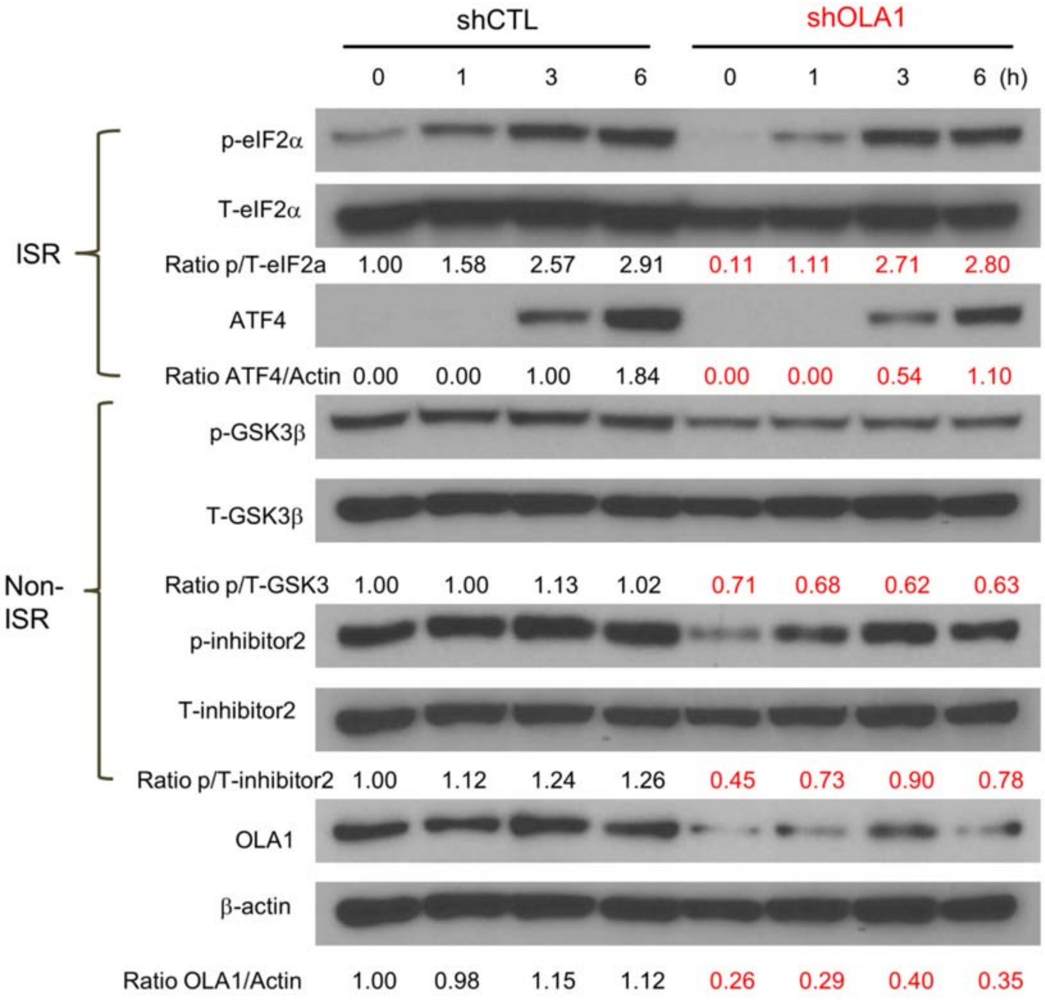

Supplementary Figure S2: OLA1 negatively regulates protein Ser/Thr dephosphorylation. SKOV3 cells stably transfected with the control (shCTL) and OLA1 shRNA (shOLA1) were treated with 2 μg/ml tunicamycin (TM) for 0, 1, 3, or 6 hours to induce ER stress, and subjected to immunoblot analysis with the indicated antibodies. β-actin was probed as a loading control, and densitometric quantification was performed using the ImageJ software.
